# Visual pigments in a living fossil, the Australian lungfish *Neoceratodus forsteri*

**DOI:** 10.1186/1471-2148-7-200

**Published:** 2007-10-25

**Authors:** Helena J Bailes, Wayne L Davies, Ann EO Trezise, Shaun P Collin

**Affiliations:** 1 School of Biomedical Sciences, University of Queensland, St Lucia, Brisbane, QLD 4072, Australia; 2 Faculty of Life Sciences, Manchester University, Oxford Road, Manchester, M13 9PL, UK; 3 UCL Institute of Ophthalmology, 11-43 Bath Street, London, EC1V 9EL, UK

## Abstract

**Background:**

One of the greatest challenges facing the early land vertebrates was the need to effectively interpret a terrestrial environment. Interpretation was based on ocular adaptations evolved for an aquatic environment millions of years earlier. The Australian lungfish *Neoceratodus forsteri *is thought to be the closest living relative to the first terrestrial vertebrate, and yet nothing is known about the visual pigments present in lungfish or the early tetrapods.

**Results:**

Here we identify and characterise five visual pigments (*rh1*, *rh2*, *lws*, *sws1 *and s*ws2*) expressed in the retina of *N. forsteri*. Phylogenetic analysis of the molecular evolution of lungfish and other vertebrate visual pigment genes indicates a closer relationship between lungfish and amphibian pigments than to pigments in teleost fishes. However, the relationship between lungfish, the coelacanth and tetrapods could not be absolutely determined from opsin phylogeny, supporting an unresolved trichotomy between the three groups.

**Conclusion:**

The presence of four cone pigments in Australian lungfish suggests that the earliest tetrapods would have had a colorful view of their terrestrial environment.

## Background

**Figure 1:**
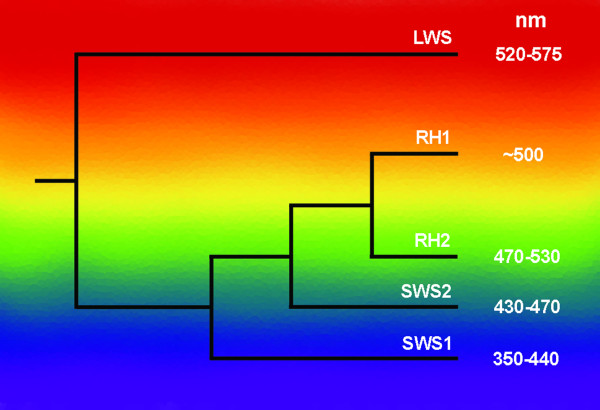
The phylogenetic relationships between the vertebrate visual opsin lineages. A series of four duplication events produced the *lws*, *sws1*, *sws2 *and then *rh1 *and *rh2 *genes. The position of each branch on the spectrum portrays the approximate spectral sensitivity of each opsin group. Maximum absorbance value ranges (nm) are based on pigments reconstructed with 11-*cis *retinal. Values are taken from [45], figure adapted from [3].

Vertebrate vision in both bright-light (photopic) and dim-light (scotopic) conditions occurs following the absorbance of light by a chromophore (based on either vitamin A_1 _or A_2_) attached to a visual pigment protein (opsin) within retinal photoreceptor cells (rods or cones). Opsin proteins are a subgroup of G-protein coupled receptors (GPCRs) with a seven transmembrane domain spanning the photoreceptor outer segment membrane. Changes in amino acids surrounding the binding pocket of the light-sensitive chromophore of the opsin can directly alter the spectral sensitivity of the visual pigment. Opsins arise from paralogous opsin genes, and the resulting visual pigments maximally absorb light from different parts of the spectrum from UV to near infrared (Fig. 1). Species from most vertebrate classes possess one or more of a total of five opsin genes; *rh1 *(medium wavelength-sensitive 1; found in rods), *rh2 *(medium wavelength-sensitive 2; found in cones), *lws *(long wavelength-sensitive; found in cones), *sws1 *(UV/violet or short wavelength-sensitive 1; found in cones), and *sws2 *(blue or short wavelength-sensitive 2; found in cones). The persistence, loss or duplication of these opsin genes reflects the spectral environment and visual needs of a species [1-3]. While the opsin complement has been well studied in a number of aquatic and terrestrial species [4-7], there remain large gaps in our understanding of the evolution of vertebrate opsins, particularly among species representing the period prior to the transition onto land such as the sarcopterygian, or lobe-finned fish.

Sarcopterygian fish gave rise to the first tetrapods and are represented today by the lungfishes (the Australian, *Neoceratodus forsteri*; the African, *Protopterus spp*.; and the South American, *Lepidosiren paradoxa*), and the coelacanth, *Latimeria chalumnae*. The relationship between all early Sarcopterygii remains controversial and highly debated despite the advent of phylogenetic analysis of nucleotide and amino acid sequences [8-10]. Some molecular analyses of sarcopterygian phylogeny reveal that the lungfish is more related to tetrapods than to the coelacanth, *L. chalumnae *[9], while others present an unresolved trichotomy between all three groups [10]. Fossil forms of the lungfish family Ceratodontidae (genus *Ceratodus*) first appear in the fossil record in the Triassic period ([11] for review). The genus *Neoceratodus *(approx. 4 species, of which *N. forsteri *is the sole survivor) is found in the fossil record from the Lower Cretaceous period 135 million years ago (mya) and therefore *N. forsteri *lays claim to being the oldest surviving vertebrate genus [12]. Consequently, the visual system of *N. forsteri *may represent an evolutionary design most closely reflecting that present just prior to the emergence of land vertebrates in the Devonian period.

The Australian lungfish *Neoceratodus forsteri *was thought to have poor eyesight due to its small eye size, low spatial resolving power [13,14], sluggish behaviour in captivity [15-17] and ability to detect prey using electroreception [18]. However, recent work on the retina of *N. forsteri *has revealed four morphologically-distinct photoreceptor types (one rod and at least three cones), some containing colored intracellular filters that are otherwise only found in terrestrial Orders [13,19]. Although a partial sequence of the African lungfish *Protopterus spp*. *rh1 *opsin gene has been previously published [[20]; Genbank: AF369054], nothing else is known about lungfish opsins. The other extant sarcopterygian fish, *L. chalumnae*, possesses only two functional opsin genes, *rh1 *and *rh2 *and lives in a photon-limited deep-sea environment [21]. Conversely, *N. forsteri *inhabits a brightly lit, shallow freshwater habitat more similar to the environment from which terrestrial evolution occurred [8]. This prompted us to investigate the complement of opsins expressed in *N*. *forsteri *in order to trace the evolution of photoreception in ancestral tetrapods.

## Results and discussion

### Opsin mRNA

We have characterised the full length cDNA coding sequences of five visual opsin genes (*rh1*, *rh2*, *lws*, *sws1 *and *sws2*), one from each of the five image-forming vertebrate opsin groups, in *N. forsteri*. In addition, 3' RACE experiments reveal multiple transcripts of *rh1 *utilising different polyadenylation (polyA) signal sequences within the rh1 3' untranslated region (UTR). All five opsins are expressed in the retina of sub-adult fish and the deduced amino acid (aa) sequences yield polypeptides ranging in size between 351–356 aa's. Lungfish opsins share highly conserved residues known to be important in visual pigment function such as a glutamate counterion at aa site 113 (numbering follows that of bovine rhodopsin [22]) and a lysine residue forming the chromophore binding site at aa site 296.

More than ten independent clones (from PCR experiments using both degenerate primers and in 3' and 5' rapid amplification of cDNA ends) from at least two individual lungfish were sequenced for each opsin gene family and, while there was evidence of polymorphism in the gene pool (and/or possible sequencing error) with a variation between clones of up to 0.5%, there was no evidence for gene duplications in individuals. We therefore conclude that we did not sequence paralogous opsins within the five main vertebrate opsin groups in *N. forsteri *and that the lungfish genome encodes a single copy of each opsin gene. This is in contrast to opsin gene duplications in teleost fish. Ray-finned fish (Actinopterygii) underwent a whole genome duplication event around 350 mya, after the divergence of the Sarcopterygii, and many lineages of teleost fish are tetraploid ([23] and references therein), whereas *N. forsteri *is a diploid animal [24]. In addition, some species of teleost fish appear to have undergone multiple opsin gene duplications independently [25]. These have accumulated subsequent amino acid changes, resulting in differences in the maximum sensitivity of opsins. These opsins can then be preferentially expressed to fine-tune the animal's spectral sensitivity to environmental light, thus reflecting a degree of visual plasticity [3,26]. For example, African cichlid fish determine their spectral sensitivity by means of preferential expression by up to seven available cone opsin genes [2,26,27].

**Figure 2:**
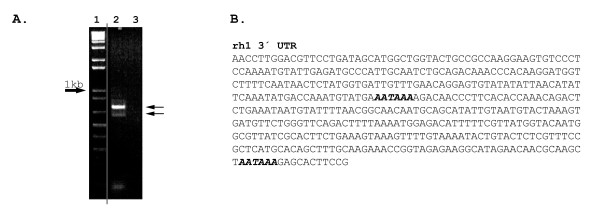
Multiple transcripts of *rh1 *occur in the Australian lungfish retina. **A**) 3' RACE PCR products from lungfish rh1 cDNA. Arrows point to two potential transcripts differing in size in lane 2 (a third, faint band is also visible above these two but was not successfully sequenced), while lane 3 contained no template cDNA. The vertical grey line indicates where an irrelevant region of gel was removed using Photoshop 6.0 (Adobe). **B**) The 3' UTR of lungfish rh1. Two sequenced polyA signals are present (bold and italicised), both of which can induce polyadenylation producing two differently sized Rh1 transcripts.

Two rh1 polyA transcripts were successfully sequenced from *Neoceratodus forsteri *(a third, faint band was also visible but not sequenced, fig. 2) and sequence analysis shows that the two polyA signals are present in a tandem array within the 3' UTR. This finding adds to increasing evidence that multiple transcripts arising from tandem polyA signals are a common, yet little studied, feature of *rh1 *genes [28-31]. The longer lungfish rh1 transcript shows a more intense band than the shorter transcripts (Fig. 2A), which could be a result of differential stability of the transcripts. There is evidence that some genes contain regulatory elements between polyA sites, which influence the stability of the longer mRNA transcript [32]. rh1 transcript levels in mice change according to circadian rhythms, possibly due to inherent transcript stability or even a circadian variation in competing mRNA processing factors and therefore site preference [33]. The use of alternative polyA transcripts as a mechanism of *rh1 *gene regulation therefore evolved early in vertebrate evolution and has been selectively maintained in mammals.

### Phylogenetic analyses

**Figure 3:**
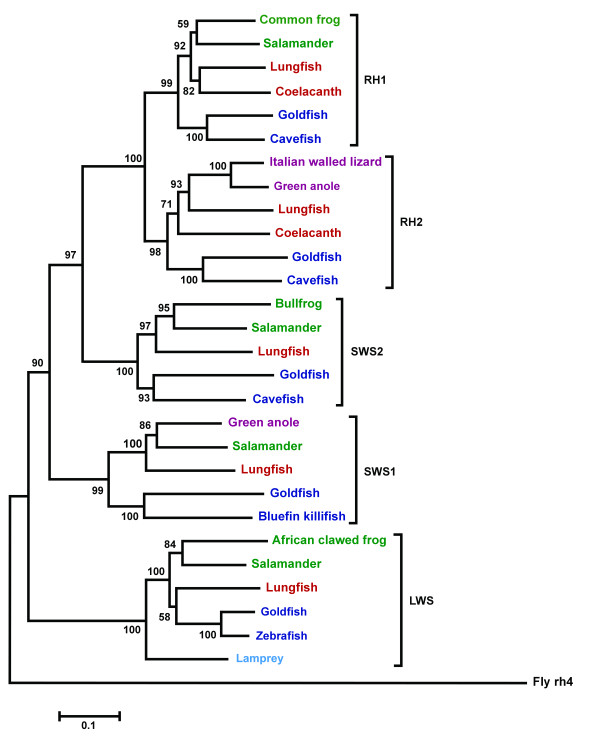
A phylogenetic tree of the five photoreceptor opsins of *Neoceratodus forsteri *and selected full-length nucleotide coding sequences of related species. The tree was constructed using the Neighbour-joining method with 1000 bootstrap replications [39]. Sarcopterygian fish (coelacanth and lungfish) are in red, agnathan fish (lamprey) are in light blue, teleost fish are in dark blue, amphibians are in green and reptiles are in purple. Genbank accession numbers are listed in Table 1. Bootstrap confidence values are at the base of each node. The rh4 opsin of *Drosophila melanogaster *(Table 1) was used as an outgroup. Scale bar indicates nucleotide substitutions per site.

**Figure 4:**
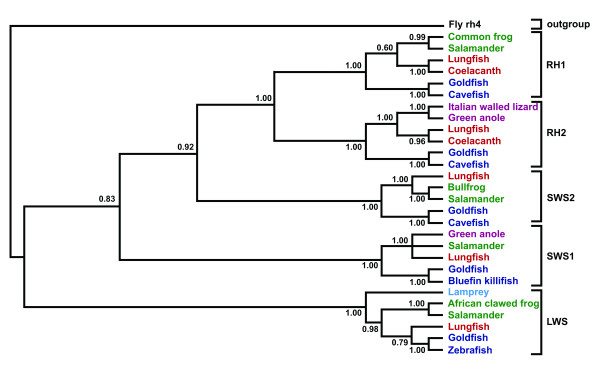
A clade credibility tree showing the relationships between lungfish and other selected vertebrate opsins. The tree was generated using Bayesian inference *via *a Metroplis-coupled Markov chain Monte Carlo simulation. Sarcopterygian fish (coelacanth and lungfish) are in red, teleost fish are in blue, amphibians are in green and reptiles are in maroon. Genbank accession numbers are listed in Table 1. Posterior probability values are at the base of each node. The rh4 opsin of *Drosophila melanogaster *is used as an outgroup (Table 1). The probability of most relationships within the tree is 1.00 after 300,000 generations, while lower posterior probability values are found within the lws and rh1 groups.

Phylogenetic comparison of codon-matched alignments of both nucleotide and deduced amino acid sequences of *rh1*, *rh2*, *lws1*, *sws1 *and *sws2 *with other vertebrate opsins (Genbank accession numbers listed in Table 1) using two fundamentally different methods of phylogenetic inference (both the Neighbour-joining (NJ) method and Bayesian inference *via *a Metropolis Markov chain Monte Carlo simulation) reveals that lungfish opsins share more similarity with tetrapod opsins than those of other fishes (Figs. 3 and 4 and Additional file 1). Lungfish opsins form a clade with tetrapod opsins rather than teleost fish in four out of the five opsin families in each analysis, but different topology is obtained between tree branches with low bootstrap values or low posterior probability, in particular within the *rh1 *and *lws *groups.

**Table 1:** Species and Genbank accession numbers [46] of opsin nucleotide sequences and deduced amino acids used in phylogenetic analyses.

Opsin	Common name reference in Fig. 3	Species	Genbank accession number
Invertebrate Rh4	Fruit Fly	*Drosophila melanogaster*	NM_057353
Rh1	Common frog	*Rana temporaria*	U59920
	Salamander	*Ambystoma tigrinum*	U36574
	Lungfish	*Neoceratodus forsteri*	EF526295
	Coelacanth	*Latimeria chalumnae*	AF131253
	Cavefish	*Astyanax fasciatus*	U12328
	Goldfish	*Carassius auratus*	L11863
Rh2	Green anole	*Anolis carolinensis*	AF134189
	Italian wall lizard	*Podarcis sicula*	AY941829
	Lungfish	*Neoceratodus forsteri*	EF526296
	Coelacanth	*Latimeria chalumnae*	AF131258
	Cavefish	*Astyanax fasciatus*	AH004622
	Goldfish	*Carassius auratus*	L11865
SWS2	Salamander	*Ambystoma tigrinum*	AF038946
	Bullfrog	*Rana catesbiana*	AB010085
	Lungfish	*Neoceratodus forsteri*	EF526299
	Goldfish	*Carassius auratus*	L11864
	Cavefish	*Astyanax fasciatus*	AH007939
SWS1	Green anole	*Anolis carolinensis*	AF134192
	Salamander	*Ambystoma tigrinum*	AF038948
	Lungfish	*Neoceratodus forsteri*	EF526298
	Goldfish	*Carassius auratus*	D85863
	Bluefin killifish	*Lucania goodei*	AY296735
LWS	African clawed frog	*Xenopus laevis*	U90895
	Salamander	*Ambystoma tigrinum*	AF038947
	Lungfish	*Neoceratodus forsteri*	EF526297
	Goldfish	*Carassius auratus*	L11867
	Zebrafish	*Danio rerio*	NM_001002443
	Lamprey	*Geotria australis*	AY366491

Phylogenetic analysis of *rh *opsin nucleotide sequences does not favour the coelacanth as a closer relative to tetrapods than lungfish and further supports an unresolved trichotomy between the coelacanth, lungfish and early tetrapods, which varies according to the gene family investigated and the method of analysis [10]. For example, the phylogenetic tree produced using the NJ method with nucleotide sequences places lungfish *rh2 *together with tetrapod opsins rather than the coelacanth opsin gene. Within the *rh1 *rhodopsin group, however, lungfish and coelacanth sequences are placed together as a sister group to the tetrapod rod pigments. Bayesian inference favours the lungfish and coelacanth forming a sister group to the tetrapods from both *rh1 *and *rh2 *sequences (Fig. 4). Conversely, comparison of amino acid sequences using the NJ method [see Additional file 1] supports coelacanth opsins forming a sister group to both teleost and tetrapod opsins, while lungfish *rh1 *and *rh2 *genes are placed as both a sister group to teleost fish opsins (*rh1*) and tetrapod opsins (*rh2*).

The fish-tetrapod transition occurred in a space of <20 million years around 400 mya [8] and a large array of genes or whole genome sequences may therefore be needed to resolve the trichotomy between sarcopterygian fish [10]. It is unfortunate that the coelacanth genome does not encode a functional sws1 pigment as *sws1 *genes have recently been proposed as a good marker for vertebrate phylogenies [34].

## The lungfish visual system

Our results demonstrate that the full complement of the orthologues of the known vertebrate photoreceptive visual pigment genes are expressed in the retina of the Australian lungfish, the nearest, and most primitive, extant relative to the land vertebrates. Partial sequences of *rh1 *and *lws *have also been successfully amplified from the more derived African lungfish *Protopterus sp. *(unpublished data, H.J. Bailes, W.L. Davies, A.E.O. Trezise and S.P. Collin). An earlier molecular study of visual pigments in the retina of a sarcopterygian fish (the coelacanth *Latimeria chalumnae*) found only two opsins, *rh1 *and *rh2 *in addition to a pseudogene *Ψsws1 *[21]. The coelacanth inhabits a photon-limited deep-sea environment and the move to this low-light, shortwave-shifted environment meant that the subsequent loss of three cone visual pigments (LWS, SWS1 & SWS2 used for color vision in bright-light environments in other vertebrates [21]) was not a selective disadvantage, revealing a correlation between loss of opsin genes and the spectral habitat/functional needs of a species [2]. Conversely, the retention of all five vertebrate opsin families in *N. forsteri *suggests that the dipnoan lineage has lived in a brightly-lit colorful environment throughout its evolutionary history, making it a more appropriate model organism for the early tetrapod visual system than the coelacanth.

## Conclusion

The characterization of four cone opsins reveals the potential for tetrachromatic vision in lungfish, although behavioral work is needed to verify if lungfish can discriminate objects based on differences in chromatic hue. Multiple opsins and a range of colored intraocular filters in the retina of *N. forsteri *[13,19] suggest that it is adapted for diurnal vision, in contrast to earlier reports that adult lungfish are crepuscular [16,35]. Lungfish have a mostly carnivorous diet [16] and may utilise color vision in prey capture or reproductive behaviour. These findings indicate that the first tetrapods probably possessed eyes adapted for chromatic diurnal vision, with all five opsins expressed in the retina. Colored oil droplets within the photoreceptors would have also filtered the incident light, enhancing color discrimination by reducing the spectral overlap of pigment absorbance curves [13,36].

## Methods

One adult lungfish was caught by hook and line in the Mary River near Tiaro, Queensland (Queensland Fisheries Management Authority Permit No. PRM01599G). Three subadult and two juvenile fish were bred in captivity and donated by Prof. Jean Joss from Macquarie University, Sydney, Australia. Animals were sacrificed using an overdose of benzocaine dissolved in acetone (according to animal ethics guidelines of the University of Queensland AEC No. ANAT/436/04/ARC). Australian lungfish are listed as 'Vulnerable' under the Australian *Commonwealth Environment Protection and Biodiversity Conservation Act 1999 *and as such only a limited number of animals were available for this work.

### Opsin mRNA

Dissected retinae were placed in RNAlater solution at 4°C. Total RNA was extracted using a Macherey-Nagel Nucleospin-RNA II kit (Machery-Nagel GmbH & Co. K.G.) for individual adult and subadult eyes, or from pooled left and right eyes for each juvenile fish used. Total RNA was converted to cDNA using Superscript II (Invitrogen Corp.) and random 9-mer or 16-mer oligo-dT primers. A series of degenerate primers were designed to conserved regions specific to each of the five vertebrate retinal opsin families (*rh1*, *rh2*, *lws*, *sws1 *and *sws2*). Primers were used in nested PCR on cDNA using standard methods [37]. Amplified fragments were cloned into pBluescript vector (Stratagene Inc.) and sequenced at the Australian Genome Research Facility (AGRF Ltd.). Once sequenced fragments were obtained and assembled, rapid amplification of cDNA ends (RACE) at both the 5' and 3' ends was performed to produce the full-length sequence of visual opsin mRNA. Specific primers were then designed to the 5' and 3' ends of each opsin and used in conjunction with a proof-reading enzyme (Phusion; Finnzymes Oy) to verify the full length coding sequence of each opsin identified (*rh1 *forward TTA GGA GCT GCA ACC ATG AAC GGA ACA GAG, *rh1 *reverse [polyA transcript1] GCT TGT GGG TTT GTC TGC AGA TTG CAA TGG, *rh1 *reverse [polyA transcript2] CCG TTC TAT GCC TTC TCT ACC GGT TTC TTG, *rh2 *forward ACC AAC AGC AGT AGT GTA TTC GCA GCA AAG, *rh2 *reverse AGG GAT ACT TGG CTT GAG GAG ACT GAA GAG, *lws *forward ATA GAG ACA GAG AGG GAG AGA TGG CTG AAC, *lws *reverse CGC CGT ACA GTC ATT GCT TGT GAA ATA GTG, *sws1 *forward AGC AGA CAG AAG ATG TCA GGG GAA GAA GAG, *sws1 *reverse GCC ATA ACA CAA CTA AGG GGC CAT CAC TTC, *sws2 *forward CCG GGT TAC ACA CCA CTA CAA GTC AAC TAC, *sws2 *reverse AAT GGC TGG AGG AGA CCG AAG AGA CCT GAG). The verification of each opsin with these primer sets was carried out using cDNA from additional individuals from those used in original opsin identification experiments. In this way, each sequence was confirmed as transcribed in at least two individuals.

### Phylogenetic analyses

Full-length coding sequences were compared against known sequences from other fish and tetrapods in the Genbank database, and against an outgroup of *Drosophila melanogaster *rh4 (Table 1). A codon-matched alignment was carried out using ClustalW (European Bioinformatics Institute) and by manual inspection. Phylogenetic analysis of nucleotides and corresponding amino acids was performed using MEGA3 [38] software and the Neighbour-joining (NJ) method [39] with 1000 bootstrap replications. The Tamura-Nei [40] model of DNA evolution was used, with complete deletion and a homogeneous pattern of nucleotide substitution among lineages and uniform rates of nucleotide substitution across all sites. The NJ method with 1000 bootstrap replications, a homogeneous pattern of substitution among lineages and uniform rates of evolution was also used to infer phylogeny from deduced amino acids using a Poisson correction substitution model. Phylogeny was also deduced using Bayesian inference *via *a Metropolis-coupled Markov chain Monte Carlo (MCMC) simulation using MrBayes 3.1 software [41,42]. A general time-reversible model (GTR [43]) of DNA evolution was used, with a gamma-shaped rate variation with a proportion of invariable sites. Two simultaneous runs were performed for 300,000 generations with chains sampled taken every 1000 generations. The first 75 trees sampled (25%) were discarded as burnin. Consensus trees were extracted in Treeview 1.6.6 [44].

## Authors' contributions

HJB carried out the PCR work, phylogenetic analysis and drafted the manuscript. WLD helped design and co-ordinate PCR work. AEOT helped with phylogenetic analysis and the design and co-ordination of the study. SPC helped with the conception, design and co-ordination of the study. All authors read, contributed to, and approved the final manuscript.

## Supplementary Material

Additional file 1 A phylogenetic tree of the five photoreceptor opsins of *Neoceratodus forsteri *and selected deduced amino acid sequences from full-length nucleotide coding sequences of related species. An additional figure representing a phylogenetic tree of lungfish opsins using deduced amino acid sequences. A phylogenetic tree of the five photoreceptor opsins of *Neoceratodus forsteri *and selected deduced amino acid sequences from full-length nucleotide coding sequences of related species. The tree was constructed using the Neighbour-joining method with 1000 bootstrap replications and a Poisson correction substitution model. Sarcopterygian fish (coelacanth and lungfish) are in red, teleost fish are in dark blue, amphibians are in green and reptiles are in purple. Genbank accession numbers are listed in Table 1. Bootstrap confidence values are at the base of each node. The rh4 opsin of *Drosophila melanogaster *(Table 1) was used as an outgroup. Scale bar indicates amino acid substitutions per site. Click here for file
